# Sirtuins and Type 2 Diabetes: Role in Inflammation, Oxidative Stress, and Mitochondrial Function

**DOI:** 10.3389/fendo.2019.00187

**Published:** 2019-03-27

**Authors:** Munehiro Kitada, Yoshio Ogura, Itaru Monno, Daisuke Koya

**Affiliations:** ^1^Department of Diabetology and Endocrinology, Kanazawa Medical University, Uchinada, Japan; ^2^Division of Anticipatory Molecular Food Science and Technology, Medical Research Institute, Kanazawa Medical University, Uchinada, Japan

**Keywords:** SIRT1, SIRT2, SIRT3, SIRT6, Type 2 diabetes

## Abstract

The rising incidence of type 2 diabetes mellitus (T2DM) is a major public health concern, and novel therapeutic strategies to prevent T2DM are urgently needed worldwide. Aging is recognized as one of the risk factors for metabolic impairments, including insulin resistance and T2DM. Inflammation, oxidative stress, and mitochondrial dysfunction are closely related to both aging and metabolic disease. Calorie restriction (CR) can retard the aging process in organisms ranging from yeast to rodents and delay the onset of numerous age-related disorders, such as insulin resistance and diabetes. Therefore, metabolic CR mimetics may represent new therapeutic targets for insulin resistance and T2DM. Sirtuin 1 (SIRT1), the mammalian homolog of Sir2, was originally identified as a nicotinamide adenine dinucleotide (NAD^+^)-dependent histone deacetylase. The activation of SIRT1 is closely associated with longevity under CR, and it is recognized as a CR mimetic. Currently, seven sirtuins have been identified in mammals. Among these sirtuins, SIRT1 and SIRT2 are located in the nucleus and cytoplasm, SIRT3 exists predominantly in mitochondria, and SIRT6 is located in the nucleus. These sirtuins regulate metabolism through their regulation of inflammation, oxidative stress and mitochondrial function via multiple mechanisms, resulting in the improvement of insulin resistance and T2DM. In this review, we describe the current understanding of the biological functions of sirtuins, especially SIRT1, SIRT2, SIRT3, and SIRT6, focusing on oxidative stress, inflammation, and mitochondrial function, which are closely associated with aging.

## Introduction

The rising incidence of type 2 diabetes mellitus (T2DM) has significantly increased worldwide in recent decades, and the development of better treatments for T2DM is urgently needed. Aging is a universal process that affects all organs. Age-related disruptions in cellular homeostasis result in the decline in the responsiveness to physiological stress, including oxidative stress and inflammation, which are implicated in the pathogenesis of metabolic diseases, including insulin resistance and T2DM. Additionally, mitochondria play a central role in energy production and responsiveness to nutrient availability, and they are one of the sources of reactive oxygen species (ROS) (1). Therefore, mitochondrial function decline is also closely related to the impairment of metabolic homeostasis (2) and oxidative stress (3, 4), contributing to the progression of insulin resistance and T2DM, which are associated with aging. Additionally, oxidative stress is closely linked to inflammation (5, 6); therefore, the suppression of oxidative stress/inflammation and preservation of mitochondrial function should be therapeutic targets for insulin resistance and T2DM, as well as for anti-aging treatments.

Calorie restriction (CR) retards aging or extends the life spans of yeast, worms, flies, and rodents (7). The benefits of CR for the suppression of age-related diseases, including glucose intolerance, cardiovascular disease and cancer, have also been observed in rhesus monkeys or humans (8–10), by improving insulin sensitivity and reducing inflammation and oxidative stress. Sirtuins have received attention for their role in modifying lifespan, especially in relation to the benefits of CR. From the initial studies on aging in yeast, silent information regulator 2 (Sir2), a nicotinamide adenine dinucleotide (NAD^+^)-dependent deacetylase, was identified as one of the possible molecules through which CR improves lifespan extension (11). Homologs of Sir2 in higher eukaryotic organisms are known as SIRT1, which may contribute to CR-induced longevity (12–14), and, currently, seven sirtuins, including SIRT1, have been identified in mammals (15, 16) (Table 1). Numerous previous reports have shown the multiple physiological roles of sirtuins, including SIRT1, SIRT2, SIRT3 and SIRT6, in cellular function, such as glucose metabolism, mitochondrial function and resistance against cellular stresses, including oxidative stress and inflammation (15–20). Thus, the modulation of sirtuin activity, as a CR mimetic, may be a novel drug target for insulin resistance and T2DM.

**Table 1:** Seven sirtuins in mammals.

**Sirtuin**	**Catalytic activity**	**Localization**
SIRT1	Deacetylase	Nucleus and cytoplasm
SIRT2	Deacetylase	Cytoplasm and nucleus
SIRT3	Deacetylase	Mitochondria
SIRT4	ADP-ribosyl transferase	Mitochondria
SIRT5	Deacetylase	Mitochondria
SIRT6	Deacetylase and ADP-ribosyl transferase	Nucleus
SIRT7	Deacetylase	Nucleus

In this review, we describe the current understanding of the biological functions of sirtuins, especially SIRT1, SIRT2, SIRT3, and SIRT6, focusing on oxidative stress, inflammation and mitochondrial function, which are closely associated with aging. We also discuss their potential as pharmacological targets to prevent the development of metabolic diseases, such as insulin resistance and T2DM.

## Inflammation, Oxidative Stress, and Mitochondrial Dysfunction, Which Are Related to the Pathogenesis of Insulin Resistance and Type 2 Diabetes

Chronic inflammation, oxidative stress and impaired mitochondrial function in skeletal muscle, adipose tissue or monocytes/macrophages (21, 22) are closely related to the pathogenesis of insulin resistance and T2DM. Additionally, inflammation and oxidative stress contribute to pancreatic β-cell dysfunction (23, 24), contributing to the progression of T2DM.

The activation of monocytes in the circulation, adipocytes and macrophages residing in adipose tissue leads to the release of various inflammatory mediators, including tumor necrosis factor-α (TNF-α), interleukin-6 (IL-6), and chemoattractant protein-1 (MCP-1), in insulin-resistant and diabetic states. These cytokines activate inflammatory signaling pathways, such as the inhibitor of IκB kinase (IKK) and c-Jun NH_2_-terminal kinase (JNK) pathways, which impair the insulin signaling pathway by modulating phosphoinositide 3-kinase (PI3K) and Akt (25–27), and they play a crucial role in the pathogenesis of insulin resistance in adipose tissue and skeletal muscle. Oxidative stress also impairs insulin signaling, which contributes to insulin resistance in T2DM. In insulin-resistant or diabetic states, in addition to hyperglycemia, other metabolites, including free fatty acids (FFAs) and certain cytokines, such as TNF-α, induce the overproduction of ROS from mitochondria. ROS trigger the activation of serine/threonine kinases, such as p38 mitogen-activated protein kinase (p38 MAPK), JNK, and IKK, which induce the serine phosphorylation of insulin receptor substrate-1 (IRS-1), and then degrade IRS-1 and reduce IRS-1 tyrosine phosphorylation, leading to the suppression of insulin signaling (28–31), as well as inflammation. Inflammatory mediators and oxidative stress are also related to pancreatic β-cell dysfunction, resulting in the impairment of insulin production or excretion from β cells (23, 24).

**Figure 1:**
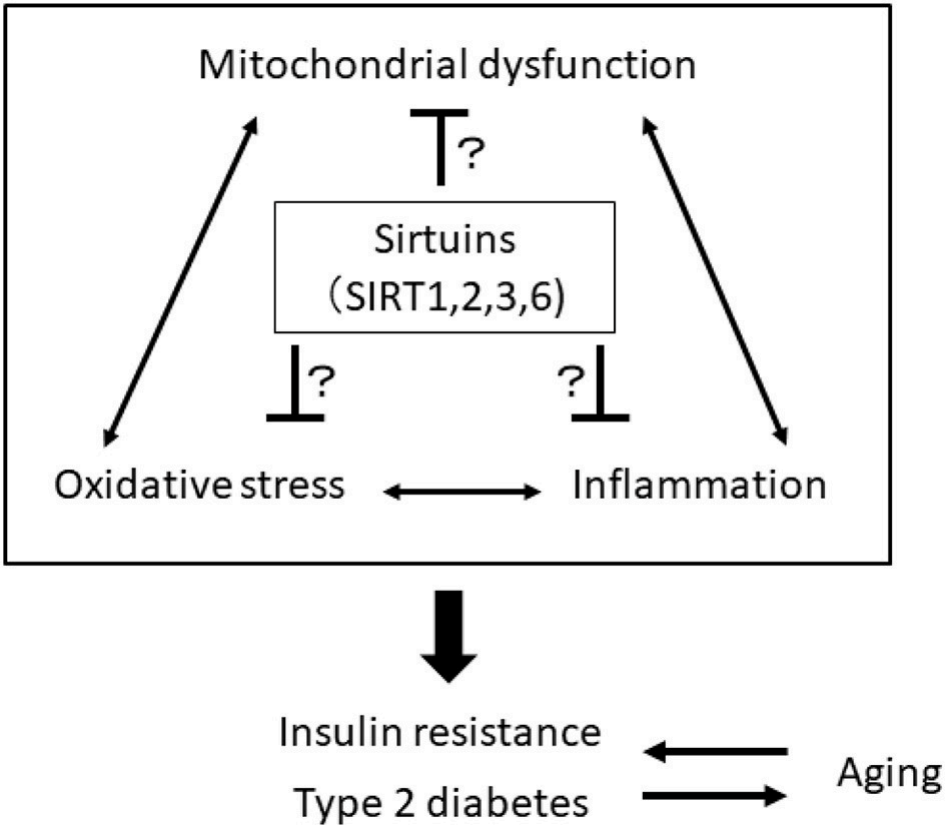
A vicious cycle among oxidative stress, inflammation, and mitochondrial dysfunction is involved in the pathogenesis of insulin resistance, type 2 diabetes, and aging. Sirtuins, including SIRT1, 2, 3, and 6, may be a therapeutic target for the treatment of age-related insulin resistance and T2DM by breaking this vicious cycle.

The impairment of mitochondrial function in skeletal muscle is involved in the pathogenesis of insulin resistance and progression of T2DM, which may be associated with aging. Mitochondria play a central role in energy production and responsiveness to nutrient availability by regulating mitochondrial oxidative phosphorylation (OXPHOS) and fatty acid oxidation. However, previous studies have shown that the rate of mitochondrial OXPHOS is reduced and that intramyocellular lipid accumulation is increased in the skeletal muscle of patients with insulin resistance and T2DM and in elderly individuals (32–35). Aging is closely linked to the impairment of metabolic homeostasis, such as insulin resistance and T2DM, which are closely related to the decline in mitochondria function. Mitochondrial function decline generates excess ROS from mitochondria, and oxidative stress is linked to inflammation. Thus, there is a vicious cycle among oxidative stress, inflammation and mitochondrial dysfunction, and breaking this cycle may be a therapeutic target for the treatment of age-related insulin resistance and T2DM, focusing on SIRT1, SIRT2, SIRT3, and SIRT6 (Figure 1).

### SIRT1

SIRT1 exists in the nucleus and cytoplasm, and it has NAD^+^-dependent deacetylase activity (16). Numerous non-histone proteins, including transcription factors, transcriptional coregulatory proteins and histones, serve as substrates for SIRT1, which is associated with a wide variety of cellular processes (16). SIRT1 may play a crucial role in reducing inflammation and oxidative stress and improving mitochondrial function, resulting in both the protection of pancreatic β cells and amelioration of insulin resistance in insulin-sensitive tissues such as skeletal muscle and adipose tissue. Therefore, SIRT1 should be a pharmacological therapeutic target to treat insulin resistance and T2DM (17, 36).

### Regulation of Inflammation

Accumulated evidence has demonstrated that SIRT1 suppresses inflammatory processes likely through interfering with nuclear factor kappa-B (NF-κB) signaling. Yeung et al. found that SIRT1 deacetylates the p65 subunit of NF-κB at lysine 310 and inhibits its transcriptional activity (37).

**Figure 2:**
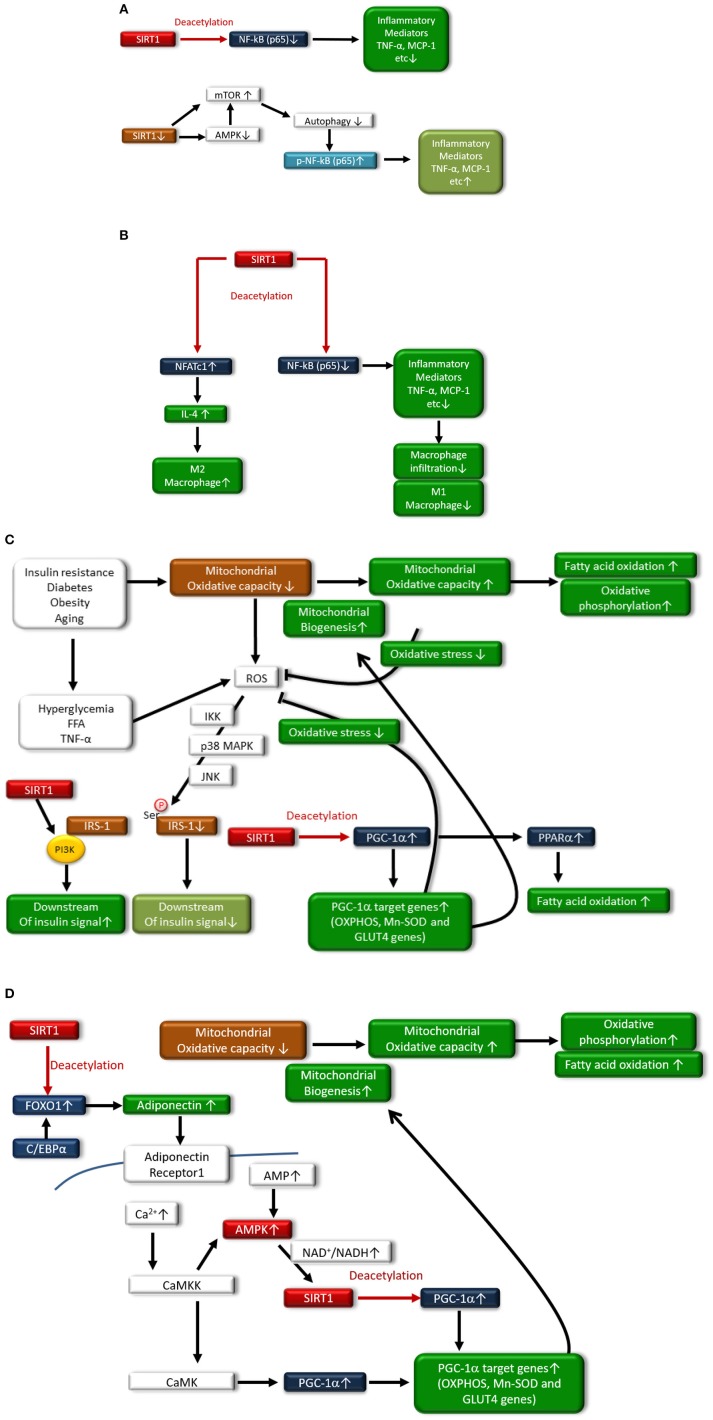
**(A)** In monocytes/macrophages and adipocytes, SIRT1 deacetylates NF-κB(p65), resulting in reduced expression of inflammatory mediators such as TNF-α and MCP-1. SIRT1 inactivation also induces inflammation through the phosphorylation of the NF-κB pathway via impaired autophagy, which is associated with activation of mammalian target of rapamycin (mTOR) and reduced activation of AMP-activated kinase (AMPK). **(B)** In adipocytes, SIRT1 deacetylates nuclear factor-κB p65 subunit [NF-κB(p65)], resulting in reduced expression of inflammatory mediators such as tumor necrosis factor-α (TNF-α) and chemoattractant protein-1 (MCP-1), and decreased polarization to M1 macrophages and infiltration to adipose tissue. SIRT also induces polarization to M2 macrophages through increased expression of interleukin-4 (IL-4) expression via deacetylation of nuclear factor of activated T-cells 1 (NFATc1). **(C)** In skeletal muscle, SIRT1 increases mitochondrial biogenesis and fatty acid oxidation through acetylation and activation of the peroxisome proliferator-activated receptor (PPAR)-γ coactivator-1α (PGC-1α). Under conditions of insulin resistance, diabetes, obesity, or aging, mitochondrial oxidative capacity is decreased, contributing to the generation of reactive oxygen species (ROS) in mitochondria. Hyperglycemia, free fatty acids (FFAs) and TNF-α stimulate ROS production from the mitochondria, and increased levels of ROS lead to the serine-phosphorylation of insulin receptor substrate-1 (IRS-1), resulting in reduced insulin signaling. However, SIRT1 interacts with phosphoinositide 3-kinase (PI3K), leading to activation of insulin signaling. Additionally, SIRT1 activates PGC-1α transcriptional activity to induce mitochondrial biogenesis and the induction of antioxidative enzymes, which can inhibit the generation of ROS by mitochondria. Expression of glucose transporter 4 (GLUT4) is enhanced through deacetylation of PGC-1α by SIRT1. Moreover, SIRT1 activates peroxisome proliferator-activated receptor-α (PPAR-α), which induces fatty acid oxidation. **(D)** SIRT1 deacetylates Forkhead box protein O1 (FOXO1) and enhances its interaction with CCAAT/enhancer binding protein α (C/EBPα), resulting in the enhanced transcription of adiponectin in adipocytes. In skeletal muscle, adiponectin is involved in the regulation of Ca^2+^ signaling and PGC-1α expression through calcium/calmodulin-dependent protein kinase kinase (CaMKK) and calcium/calmodulin-dependent protein kinase (CaMK) activation. Adiponectin activates SIRT1 through AMPK activation, thereby deacetylating PGC-1α and resulting in mitochondrial biogenesis, increased fatty acid oxidation, and oxidative phosphorylation.

In adipocytes and macrophages, SIRT1 reduces inflammatory process through the deacetylation of NF-κB (p65), leading to improved glucose metabolism (38, 39) (Figure 2A). Additionally, myeloid cell-specific SIRT1 knockout (KO) mice that were fed a high-fat diet (HFD) exhibited macrophage activation and elevated expression of inflammatory mediators in the liver and adipose tissues, which was associated with the development of systemic insulin resistance (40). Furthermore, we previously reported another mechanism regarding SIRT1 inactivation-induced inflammation (Figure 2A): SIRT1 inactivation may enhance the NF-κB signaling pathway by the phosphorylation of NF-κB (p65) via the cellular accumulation of p62/Sqstm1 due to autophagy dysfunction, in THP-1 cells, cultured human monocytes (41). Moreover, SIRT1 inactivation resulted in increased activation of the mammalian target of rapamycin complex 1 (mTORC1) pathway and reduced 5′ AMP-activated kinase (AMPK) activation, possibly contributing to impairment in autophagy (41). In humans, reduction of SIRT1 expression levels in circulating monocytes is correlated with glucose intolerance, insulin resistance and metabolic syndrome in humans (42). Gillum et al. also demonstrated that SIRT1 expression is decreased in adipose tissues from obese males and that the mRNA expression of CD14, a macrophage marker, in adipose tissue is negatively correlated with SIRT1 expression (43).

Interestingly, adipocyte SIRT1 controls systemic glucose homeostasis and insulin sensitivity through cross talk with adipose-resident macrophages (44) (Figure 2B). Hui et al. recently reported that adipose-specific SIRT1 KO mice showed a higher susceptibility to HFD-induced insulin resistance, which is associated with an increased number of adipose-resident macrophages and their polarization toward the proinflammatory M1 subtype, overexpressing inflammatory mediators (44). SIRT1 in adipocytes modulates the expression and secretion of several adipokines, including adiponectin, MCP-1, TNF-α, and IL-4, which, in turn, alters the recruitment and polarization of macrophages in adipose tissues. In adipocytes, SIRT1 enhances IL-4 expression through deacetylating the transcription factor nuclear factor of activated T cells, cytoplasmic 1 (NFATc1), leading to polarization of the M2 subtype (44). Thus, SIRT1 may diminish inflammation in adipose tissues and monocytes/macrophages and may improve insulin resistance and T2DM.

In addition, SIRT1 protein expression is reduced in skeletal muscle and primary myotubes derived from T2DM patients, suggesting that diminished SIRT1 contributes to insulin resistance induced by TNF-α in skeletal muscle (45). SIRT1 interacts with PI3K adaptor subunit p85 in an insulin-independent manner and activates insulin signaling at physiological insulin concentrations in skeletal muscle cells (45) (Figure 2C). Furthermore, SIRT1 protects pancreatic β cells against various toxic stresses, including oxidative stress and inflammatory cytokines, through suppressing NF-κB signaling (46).

### Regulation of Mitochondrial Function

SIRT1 can be involved in the regulation of metabolism and insulin resistance through the modulation of mitochondrial function. Peroxisome proliferator-activated receptor-γ coactivator-1α (PGC-1α) maintains mitochondrial biogenesis and OXPHOS proteins, leading to efficient β oxidation of fatty acids in skeletal muscle. However, the levels of PGC-1α in skeletal muscle are decreased in T2DM. SIRT1 regulates mitochondrial function and metabolic homoeostasis, increases the oxygen consumption in skeletal muscle and leads to the expression of OXPHOS genes and mitochondrial biogenesis through the deacetylation of PGC-1α. SIRT1 knockdown largely prevents the upregulation of PGC-1α-induced genes that are involved in mitochondrial fatty acid utilization (47). Furthermore, SIRT1 can regulate peroxisome proliferator-activated receptor-α (PPAR-α) activation by PGC-1α deacetylation, resulting in increased fatty acid oxidation. Thus, the activation of SIRT1 may improve insulin resistance through promoting fatty acid oxidation and mitochondrial biogenesis via deacetylation of PGC-1α and PPAR-α activation in skeletal muscle (Figure 2C). Additionally, PGC-1α remarkably increases the expression of glucose transporter 4 (GLUT4) and activation of glucose transport in murine C2C12 myotubes (48). The effects of PGC-1α on GLUT4 gene expression leads to increased transport of glucose in myocytes, suggesting that the activation of PGC-1α by SIRT1 is involved in insulin sensitization (Figure 2C). Adiponectin has antidiabetic power (49), and the levels of plasma adiponectin are reduced in insulin resistance and T2DM (50, 51). Treatment with adiponectin can decrease glucose levels and ameliorate insulin resistance in mice (52). Mechanistically, adiponectin enhances insulin sensitivity through increasing fatty acid oxidation via AMPK and PPAR-α activation (49). Additionally, SIRT1 deacetylates Forkhead box protein O1 (FOXO1) and enhances its interaction with CCAAT/enhancer binding protein α (C/EBPα), resulting in enhanced transcription of the adiponectin gene in adipocytes (53) (Figure 2D). Iwabu et al. demonstrated that adiponectin signaling plays a crucial role in skeletal muscle cells and is implicated in the regulation of Ca^2+^ signaling and expression/activation of PGC-1α in muscle adiponectin receptor (adipoR) 1 KO mice (54). Adiponectin activates AMPK by binding to adipoR1, thereby activating SIRT1, and deacetylating PGC-1α improves oxidative stress, mitochondrial function, glucose/lipid metabolism, and exercise endurance (54) (Figure 2D), resulting in improved insulin resistance and T2DM. Thus, SIRT1-induced PGC-1α deacetylation leads to the improvement of mitochondrial function via mitochondrial biogenesis and induction of GLUT4 and adiponectin, demonstrating the beneficial effects against insulin resistance and T2DM.

### Regulation of Oxidative Stress

In addition to mitochondrial function modulation, PGC-1α deacetylation by SIRT1 reduces oxidative stress through the overexpression of antioxidative enzymes, including manganese-superoxide dismutase (Mn-SOD) (55). Additionally, Forkhead box protein O3a (FOXO3a) is deacetylated by SIRT1 and translocates to the nucleus, resulting in the upregulation of other antioxidative enzymes and catalases and protection against oxidative stress (56). Thus, SIRT1 may improve insulin resistance and T2DM possibly through reducing oxidative stress (Figure 2C), inducing mitochondrial biogenesis, and increasing mitochondrial function.

### SIRT2

SIRT2 is localized in both the cytoplasm and nucleus, and it is widely expressed in various tissues, including the brain, muscle, pancreas, liver, kidney, and adipose tissues. SIRT2 interacts with many histone and non-histone protein substrates, including tubulin and histone H4 (18). SIRT2 is involved in multiple cellular functions, including genomic integrity, cell growth, differentiation, and energy metabolism, and reduced SIRT2 activity has been implicated in cancer, neurodegeneration and metabolic diseases (18). Previous studies have demonstrated that SIRT2 plays an important role in various physiological processes in maintaining metabolic homeostasis, including inflammation, oxidative stress and mitochondrial function, as well as adipocyte differentiation, fatty acid oxidation, gluconeogenesis, and insulin sensitivity (18). A few reports have shown that SIRT2 exerts anti-inflammation and antioxidative stress effects and improves mitochondrial function in metabolic-related tissues, such as skeletal muscle.

### Regulation of Inflammation

SIRT2 regulates inflammation by deacetylating the NF-κB p65 subunit (57), similar to SIRT1. Pais et al. demonstrated that SIRT2 plays a crucial role as a major inhibitor of microglia-mediated inflammation and neurotoxicity through the deacetylation of NF-κB (p65) (58) (Figure 2A). In other experimental inflammatory disease models, the anti-inflammatory effect of SITR2 has been demonstrated through the suppression of the NF-κB signaling pathway (59, 60). However, further studies are necessary to elucidate whether this anti-inflammatory effect of SIRT2 may be exerted in metabolic diseases, including insulin resistance and T2DM.

### Regulation of Oxidative Stress

**Figure 3:**
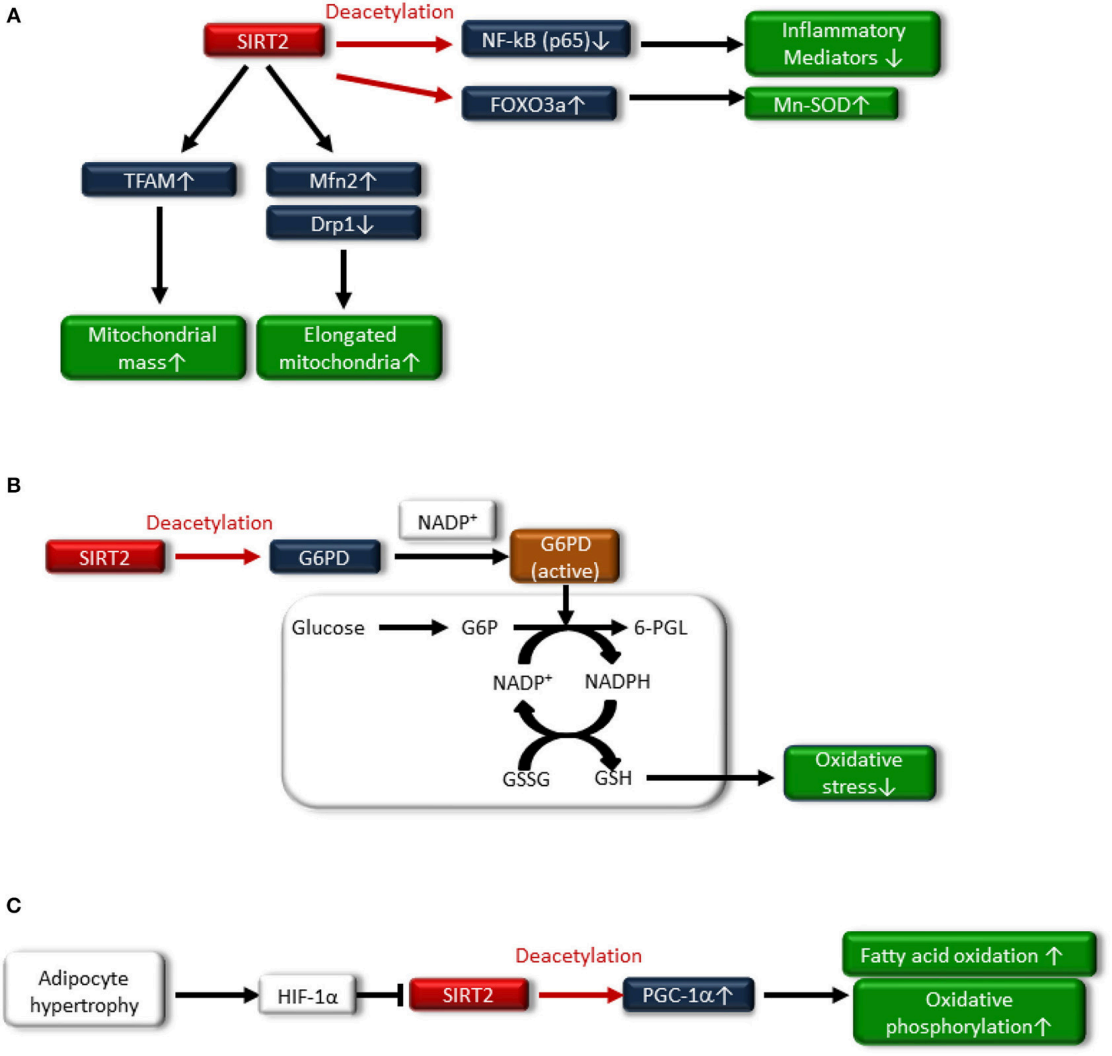
**(A)** SIRT2 deacetylates nuclear factor-κB p65 subunit [NF-κB (p65)], resulting in decreased expression of inflammatory mediators. Sirt2 also induces Mn-SOD expression by deacetylating Forkhead box protein O3a (FOXO3a). Additionally, SIRT2 increases fusion-related protein mitofusion2 (Mfn2) and decreases mitochondrial-associated dynamin-related protein 1 (Drp1), resulting in an increased number of elongated mitochondria and improved mitochondrial function. SIRT2 also attenuates the downregulation of transcription factor A, mitochondrial (TFAM), a key mitochondrial deoxyribonucleic acid (mtDNA)-associated protein, leading to an increase in mitochondrial mass. **(B)** Glucose-6-phosphate dehydrogenase (G6PD) plays an important role in the oxidative stress response by producing nicotinamide adenine dinucleotide phosphate (NADPH) and the reduced form glutathione (GSH), which is associated with deacetylating G6PD and binding to nicotinamide adenine dinucleotide phosphate (NADP^+^). **(C)** Hypoxia-inducible factor1α (HIF1α), which is accumulated in the adipocytes of hypertrophy, represses SIRT2 expression, resulting in decreased deacetylation of PGC-1α and the expression of β-oxidation and mitochondrial genes.

SIRT2 regulates redox homeostasis in cells. SIRT2-dependent deacetylation of FOXO3a leads to increased expression of Mn-SOD to improve oxidative stress (61) (Figure 3A). In addition, glucose-6-phosphate dehydrogenase (G6PD) is a key enzyme in the pentose phosphate pathway (PPP) and plays a crucial role in the oxidative stress response by producing nicotinamide adenine dinucleotide phosphate (NADPH) and reduced form glutathione (GSH), the main intracellular reductant (Figure 3B). Wang et al. reported that SIRT2 activates G6PD through deacetylation on lysine 403 in G6PD, which plays an important role in maintaining the cellular redox status and protecting cells from oxidative damage (62).

### Regulation of Mitochondrial Function

SIRT2 may be related to the regulation of mitochondrial function. Lemos et al. showed that SIRT2 is downregulated in insulin-resistant hepatocytes and the liver, and is accompanied by increased ROS production, activation of extracellular signal-regulated kinase (ERK1/2), and mitochondrial dysfunction in ob/ob mice (63). SIRT2 overexpression in insulin-resistant hepatocytes improved insulin sensitivity and reduced ROS production. SIRT2 might increase fusion-related protein mitofusion 2 (Mfn2) and decrease mitochondrial-associated dynamin-related protein 1 (Drp1), resulting in an increased number of elongated mitochondria and improving mitochondrial function (Figure 3A). SIRT2 also attenuated the downregulation of transcription factor A mitochondrial (TFAM), a key mitochondrial deoxyribonucleic acid (mtDNA)-associated protein, leading to an increase in the mitochondrial mass (63) (Figure 3A). Furthermore, SIRT2 expression in peripheral blood mononuclear cells (PBMCs) from human subjects was negatively correlated with obesity, insulin resistance and oxidative stress (63).

SIRT2 is most markedly expressed in adipocytes (61). Nutrient overload-induced adipose expansion enhances intra-adipose hypoxia, promoting the accumulation of adipocyte hypoxia-inducible factor 1α (HIF1α). HIF1α suppresses SIRT2 transcription through interaction at a cross-species conserved hypoxic response element (HRE) on the SIRT2 promoter. HIF1α accumulation in the adipocytes of human obese subjects correlates with low levels of SIRT2 in visceral adipose tissue, and reduced SIRT2 activity directly translates into decreased deacetylation of PGC-1α and expression of β-oxidation and mitochondrial genes. HIF-1α suppresses fatty acid catabolism in mitochondria by negatively regulating the SIRT2-PGC-1α axis (64) (Figure 3C).

### SIRT3

Preservation of mitochondrial health is crucial to prevent insulin resistance and T2DM during aging. SIRT3 is localized primarily in the mitochondria. SIRT3 is a major mitochondrial deacetylase and plays a major role in deacetylating and modifying the enzymatic activities of several mitochondrial proteins (16). In humans, a polymorphism in the SIRT3 gene has been correlated with reduced enzymatic efficiency and the development of metabolic syndrome (65). SIRT3 is also recognized an anti-aging molecule, and high SIRT3 expression levels are associated with longevity in humans (66, 67). Previous studies have demonstrated that SIRT3 protects organisms against metabolic stress, cancer, the development of cardiac hypertrophy, and oxidative stress (16).

### Regulation of Mitochondrial Function and Oxidative Stress

**Figure 4:**
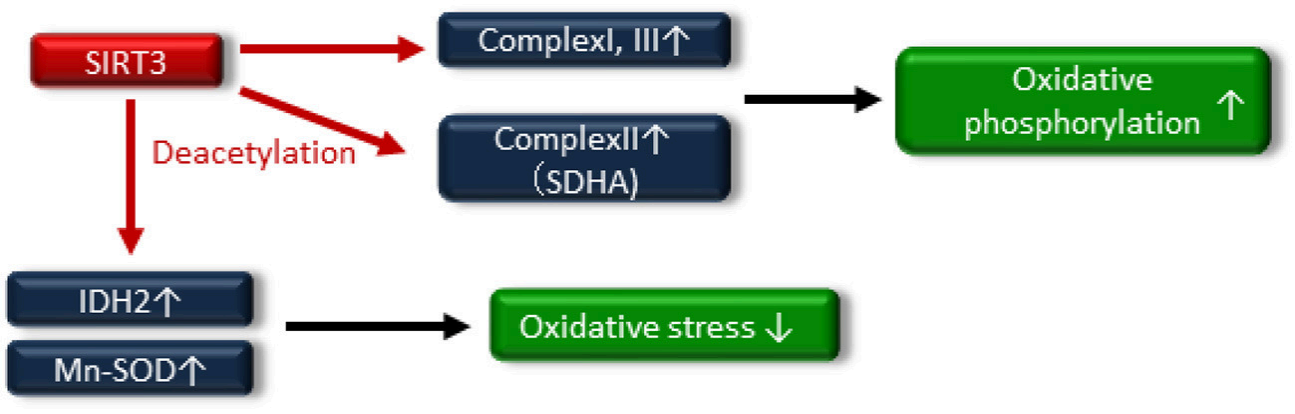
SIRT3 deacetylates the electron transport chain complexes I, II [deacetylate succinate dehydrogenase (SDH) A] and III, leading to increased oxidative phosphorylation. SIRT3 also attenuates oxidative stress by enhancing the glutathione antioxidant defense system via deacetylation and activation of isocitrate dehydrogenase 2 (IDH2) and manganese superoxide dismutase (Mn-SOD).

Numerous reports have exhibited that SIRT3 regulates mitochondrial function and maintains redox homeostasis; therefore, the impairment of SIRT3 function is implicated in the pathogenesis of insulin resistance and T2DM. Jing et al. demonstrated that decreased levels of SIRT3 in the skeletal muscle of streptozotocin (STZ)-induced diabetic mice and high-fat diet-induced obese mice were an important component of the pathogenesis of T2DM (68). SIRT3 KO mice exhibited decreased oxygen consumption and increased oxidative stress due to mitochondrial dysfunction via the hyperacetylation of complex I and III in OXPHOS, and these factors led to JNK activation and impaired insulin signaling. In addition, SIRT3 can directly deacetylate succinate dehydrogenase (SDH), a subunit of complex II and that succinate dehydrogenase activity is reduced in SIRT3 KO cells and brown adipose tissue (BAT) from SIRT3 KO mice (69). Thus, SIRT3 may induce mitochondrial oxidative phosphorylation through the deacetylation of complex I, III and SDHA of complex II (Figure 4).

A reduction in SIRT3 activity contributes to mitochondrial oxidative stress through decreasing the activation of antioxidative enzymes, such as isocitrate dehydrogenase 2 (IDH2) and Mn-SOD (70–72), by increasing the acetylation of antioxidative enzymes (Figure 4). SIRT3 protects pancreatic β cells against lipotoxicity by antagonizing oxidative stress-induced cell damage. Zhou et al. demonstrated that HFD feeding caused elevated oxidative stress accompanied by reduced SIRT3 expression in the pancreatic β cells of wild-type mice (73). Primary pancreatic islets of SIRT3 KO mice and murine pancreatic β-cell line MIN6 cells with downregulated SIRT3 expression showed increased Mn-SOD acetylation and reduced glucose-stimulated insulin secretion and glucose-stimulated ATP generation (73). On the other hand, SIRT3 overexpression, using an adenoviral system, ameliorated palmitate-induced β-cell dysfunction including endoplasmic reticulum (ER) stress in pancreatic β-cell line NIT1 cells (74). In human, reduced expression of SIRT3 in pancreatic β cells from T2DM patients has been linked to impaired β-cell function (75). Thus, novel therapeutic approaches targeting SIRT3 activity may be important in providing new opportunities to treat insulin resistance and T2DM through maintaining mitochondrial health.

### SIRT6

**Figure 5:**
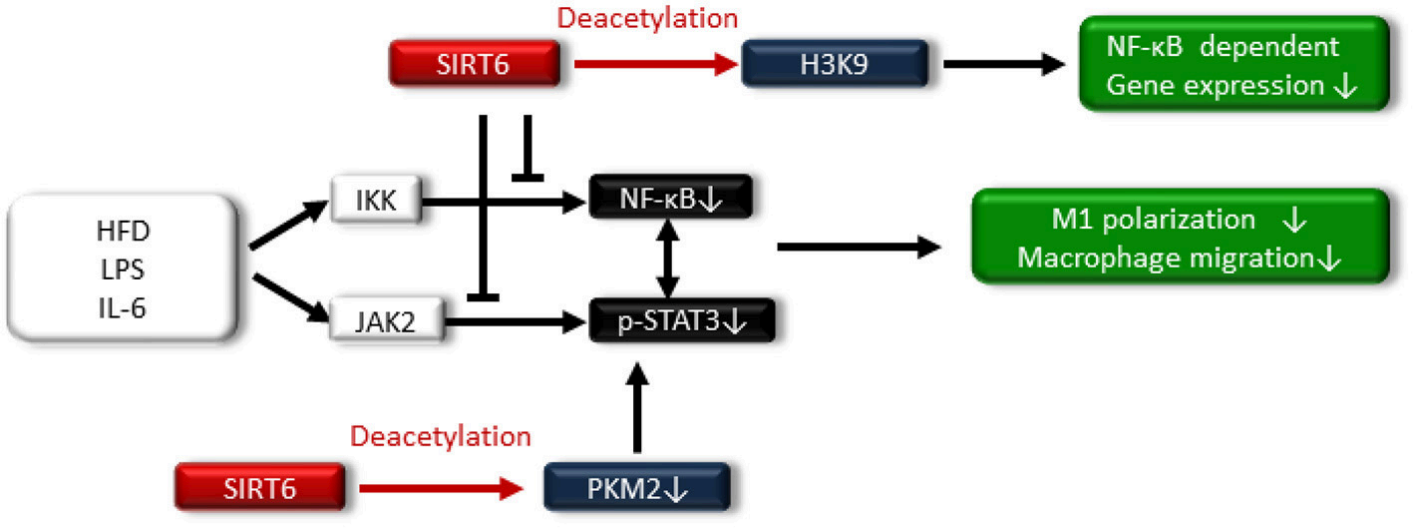
SIRT6 also attenuates NF-κB signaling via histone H3K9 deacetylation at the chromatin level. SIRT6 suppresses the high-fat diet (HFD)-, LPS-, and IL-6-induced I-κ kinase (IKK)-nuclear factor-κB (NF-κB) pathway and Janus activating kinase 2 (JAK2)-signal transducer and activator of transcription 3 (STAT3) pathway, resulting in reduced M1 macrophage polarization and macrophage migration. Additionally, SIRT6 deacetylates pyruvate kinase M2 (PKM2), preventing STAT3 from phosphorylation.

SIRT6 is located in the nucleus, and it acts as an adenosine diphosphate (ADP)-ribosyl transferase and NAD^+^-dependent deacetylase (16). SIRT6 has been associated with longevity regulation. Kanfi et al. reported that the overexpression of SIRT6 extended the lifespan of male mice and was involved in decreased serum levels of insulin-like growth factor (IGF)-1 and increased levels of IGF-binding protein 1 (76). SIRT6 is implicated in DNA repair, telomere maintenance, genomic stability and cell senescence. SIRT6 also attenuates NF-κB signaling via histone H3K9 deacetylation at the chromatin level (77–79) (Figure 5).

### Regulation of Inflammation, Oxidative Stress, and Mitochondrial Function

SIRT6 is involved in vascular inflammation and oxidative stress. Knockdown of SIRT6 in human umbilical vein endothelial cells (HUVECs) increases the expression of proinflammatory cytokines (80). Balestrieri et al. demonstrated that SIRT6 protein expression in atherosclerotic lesions of T2DM patients was downregulated, which was compared with SIRT6 protein expression in atherosclerotic lesions of non-diabetic patients, and the reduced SIRT6 expression was associated with increased oxidative stress and inflammation (81). Additionally, apolipoprotein E-deficient with SIRT6 knockdown using small hairpin RNA (shRNA) lentivirus-injected mice fed a high-cholesterol diet showed the promotion of atherosclerosis that was associated with increased inflammation in endothelial cells (82).

Regarding the relationship between SIRT6 and metabolic disease, SIRT6 levels increase in rats under the CR condition, and transgenic mice that overexpress SIRT6 are protected against HFD-induced several metabolic impairments, including glucose intolerance (83). By contrast, ablation of neural SIRT6 leads to obesity (84). Lee et al. demonstrated that myeloid-specific SIRT6 KO mice exhibit tissue inflammation and insulin resistance when fed an HFD (85). Myeloid SIRT6 deletion promoted proinflammatory M1 polarization of bone marrow macrophages and enhanced the migration potential of macrophages toward adipose-derived inflammatory mediators. SIRT6 deletion in macrophages facilitated activation of the IKK-NF-κB pathway and endogenous production of IL-6, leading to activation of the Janus activating kinase 2 (JAK2)-signal transducer and activator of transcription 3 (STAT3) pathway and positive feedback circuits for NF-κB stimulation; this cross talk expedited M1 polarization (85) (Figure 5). Furthermore, acetylated pyruvate kinase M2 (PKM2) phosphorylates STAT3 in the nucleus (86), PKM2 is deacetylated by SITR6 (87), and the study demonstrated that SIRT6 deacetylates PKM2, preventing STAT3 from being phosphorylated and leading to the suppression of M1 polarization in SIRT6-overexpressed intraperitoneal macrophages treated with LPS (Figure 5).

SIRT6 is involved in the regulation of mitochondrial function in skeletal muscle. Cui et al. demonstrated that muscle-specific SIRT6 KO mice impairs glucose homeostasis and insulin sensitivity, attenuates whole-body energy expenditure, and weakens exercise performance. Mechanistically, the deletion of SIRT6 in muscle decreased the expression of genes associated with glucose and lipid uptake, fatty acid oxidation, and mitochondrial OXPHOS in muscle cells caused by the reduced AMPK activity (88).

In pancreatic β cells, SIRT6 regulates insulin secretion in response to glucose stimulation. Xiong et al. demonstrated that the deletion of SIRT6 in pancreatic β-cells in mice leads to the impairment of glucose-stimulated insulin secretion (89, 90) and they further found that SIRT6 regulates insulin secretion by maintaining mitochondrial function and modulating intracellular Ca^2+^ dynamics (89, 90). Additionally, SIRT6 plays an important role in the protection of pancreatic β cells from lipotoxicity (palmitic acid, PA)-induced cellular dysfunction or even cell death (90). Oxidative stress generated by fatty acid oxidation is involved in the pathogenesis of PA-induced β-cell dysfunction and apoptosis (91). SIRT6 may exert the effect of antioxidative stress by coactivating NF-E2-related factor 2 (NRF2) (92). However, it is unclear whether SIRT6 plays a role in antioxidative stress in pancreatic β cells.

Thus, SIRT6 may have beneficial effects on glucose metabolism, including insulin resistance and T2DM, by reducing inflammation and improving mitochondrial function. Additionally, SIRT6 protects pancreatic β cells from lipotoxicity-induced cellular damage, through maintaining mitochondrial function and possibly antioxidative stress.

## Concluding Remarks

The knowledge of sirtuins has expanded from the original description of a NAD^+^-dependent deacetylase responsible for longevity in yeast, which is associated with CR. As described above, sirtuin family members, such as SIRT1, 2, 3, and 6, may induce beneficial effects in glucose metabolism, partially through improving inflammation, oxidative stress, and maintaining mitochondrial function. Therefore, pharmacological modulation of sirtuins may represent a novel therapeutic tool to improve insulin resistance and T2DM. Among the sirtuins, several SIRT1 activators, such as resveratrol and synthesized activators, have been evaluated for their antidiabetic effects in animal models (93). In humans, several small trials have shown that SIRT1 activators exert beneficial effects on glucose metabolism and insulin resistance, which resemble the effect of CR (94). However, there is still insufficient clinical data regarding the effect of SIRT1 activators on insulin resistance and T2DM. In addition, SIRT2, SIRT3, and SIRT6, which are induced by CR, play crucial roles in regulating cellular processes, including metabolism, inflammation, oxidative stress and mitochondrial function. However, further investigation into the targets and functions of SIRT1, SIRT2, SIRT3, and SIRT6 will aid in the development of new strategies to treat insulin resistance and T2DM. In addition to SIRT1, SIRT2, SIRT3, and SIRT6, other sirtuins, such as SIRT4, SIRT5, and SIRT7, play crucial roles in cellular homeostasis and functions, including redox homeostasis, anti-inflammation, cell survival, and mitochondrial quality control (95–100), which may be involved in the pathogenesis of insulin resistance and T2DM. However, further basic studies are necessary to elucidate the detailed molecular mechanisms.

## Author Contributions

MK and DK designed the manuscript, wrote and edited the manuscript. YO, IM, and DK contributed to the discussion. DK is the guarantor of this work.

### Conflict of Interest Statement

The authors declare that the research was conducted in the absence of any commercial or financial relationships that could be construed as a potential conflict of interest.
